# O Custo do Atraso: Disparidades Socioeconômicas e Falhas Diagnósticas no Tratamento de Infarto do Miocárdio com Oclusão

**DOI:** 10.36660/abc.20240311

**Published:** 2024-08-02

**Authors:** José Nunes de Alencar, Jesse T. T. McLaren

**Affiliations:** 1 Instituto Dante Pazzanese de Cardiologia São Paulo SP Brasil Instituto Dante Pazzanese de Cardiologia, São Paulo, SP – Brasil; 2 University Health Network Emergency Department Toronto Ontario Canadá University Health Network - Emergency Department, Toronto, Ontario – Canadá

**Keywords:** Infarto do Miocárdio, Síndrome Coronariana Aguda, Terapêutica

O infarto agudo do miocárdio com oclusão coronariana aguda representa a faceta mais grave e urgente das síndromes coronarianas agudas. Estudos seminais nos ensinaram que o restabelecimento do fluxo coronariano em pacientes com artérias ocluídas, seja por trombólise ou angioplastia primária, pode alterar a história natural da doença e reduzir significativamente a mortalidade associada a esta síndrome.^1,2^

Os autores de “O Impacto Clínico e Econômico do Atraso na Terapia de Reperfusão: Evidências do Mundo Real” fornecem dados convincentes de uma próspera região metropolitana no Brasil, concluindo que cada hora adicional de atraso na terapia de reperfusão foi associada a um aumento de 6,2% (intervalo de confiança de 95%: 0,3% a 11,8%, p = 0,032) no risco de mortalidade hospitalar.^3^ Além disso, os custos gerais foram 45% maiores entre os pacientes tratados após 9 horas em comparação com aqueles tratados nas primeiras 3 horas, principalmente devido aos custos hospitalares (p = 0,005). Gioppatto et al.^3^ também observaram, a partir de outros estudos, os impactos financeiros em pacientes e familiares devido à reperfusão atrasada e indicam a necessidade de redes de infarto do miocárdio com supradesnivelamento do segmento ST (IAMCSST) para abordar a distribuição desigual de centros com capacidade para intervenção coronária percutânea (ICP).

No entanto, gostaríamos de acrescentar duas questões a essa equação e, em um exercício de imaginação, extrapolar dois grupos de pacientes que provavelmente apresentariam resultados ainda piores do que aqueles descritos nesse artigo intrigante.

Em primeiro lugar, o nível socioeconômico mais baixo, definido como baixa renda e escolaridade inferior ao ensino secundário, é um fator determinante nas desigualdades no atendimento, conduzindo a desfechos desfavoráveis de saúde e a expectativa de vida reduzida. Estudos realizados na Suécia, Finlândia, Canadá e Estados Unidos demonstraram que o prognóstico de pacientes de grupos de nível socioeconômico mais baixo é pior após infarto agudo do miocárdio, devido a desigualdades no atendimento.^4^ Um estudo revelou que pacientes com infarto do miocárdio no grupo com renda familiar mediana mais baixa nos Estados Unidos tinham menos probabilidade de serem submetidos à angiografia coronária e à ICP.^5^ Em outras palavras, existe um acesso desigual ao atendimento, mesmo dentro dos centros de ICP, o que agrava a distribuição desigual de centros com capacidade para ICP. Como resultado, as lacunas socioeconômicas colocam os pacientes em risco de reperfusão atrasada, o que cria impactos financeiros adicionais sobre aqueles com renda menor. É importante considerar o fato de que existem disparidades econômicas mais graves no Brasil do que nos demais países mencionados neste parágrafo.

Existe um segundo grupo de pacientes que recebem reperfusão atrasada mesmo em centros com capacidade para ICP, com atraso adicional baseado na variação geográfica, ou seja, aqueles que são falsos negativos dentro do atual paradigma diagnóstico de “infarto do miocárdio com e sem supradesnivelamento do segmento ST (IAMCSST/IAMSSST). No atual paradigma diagnóstico de infarto do miocárdio, mais da metade dos pacientes com oclusão coronariana aguda (que, portanto, justificam a reperfusão imediata da artéria ocluída dentro dos tempos porta-balão ou porta-agulha)^6^ não apresentam supradesnivelamento do segmento ST e são diagnosticados com IAMSSST.^7^ Em outras palavras, esses pacientes apresentam infarto do miocárdio com oclusão, ou “oclusão coronariana aguda” (OCA), mas são falsos negativos para IAMCSST ou IAMCSST(-)OCA. Sob o paradigma atual, esses pacientes, infelizmente, recebem reperfusão atrasada em centros com capacidade para ICP, frequentemente muito além das 9 horas discutidas nesse artigo. Não apenas essas oclusões são perdidas na chegada, mas esses falsos negativos não são reconhecidos, mesmo em retrospectiva, porque o diagnóstico de alta permanece “IAMSSST”.^8^ Como resultado, esses pacientes de alto risco com IAMCSST(-)OCA não são incluídos em bases de dados de STEMI e não são considerados um alvo para melhoria da qualidade.

Vale notar que Gioppatto et al.^3^ não incluíram esses pacientes em seu estudo, justamente porque os autores optaram por selecionar apenas pacientes com resultados de testes positivos (positivos para IAMSSST) e não aqueles com doença real (OCA). Estudos demonstraram que os desfechos desses pacientes continuam a ser piores do que aqueles que têm a sorte de serem verdadeiramente positivos. Em uma metanálise de 2018 com mais de 60.000 pacientes com IAMSSST, 34% tinham uma artéria culpada ocluída com menor fração de ejeção, maior risco de choque cardiogênico, infarto do miocárdio recorrente e morte.^9^ Herman et al.^10^ compararam IAMSSST-OCA com IAMCSST-OCA e verificaram uma taxa de risco de 1,84 para mortalidade em 1 ano e 2,59 para mortalidade em 5 anos, com uma diferença absoluta de mortalidade de 15%. O tempo médio para intervenção foi de 1,4 horas no grupo com IAMCSST e 16,3 horas no grupo com IAMSSST-OCA.^10^

O estudo de Gioppatto et al.^3^ é um marco significativo na cardiologia brasileira ao destacar fortemente a necessidade urgente de realizar reperfusão o mais rápido possível para evitar desfechos desfavoráveis a nível individual e coletivo. Acrescentamos que a comunidade de cardiologia brasileira também deveria se focar em dois grupos frequentemente negligenciados com OCA, aos quais é negado tratamento oportuno e apropriado: aqueles com nível socioeconômico mais baixo, que recebem reperfusão atrasada, mesmo quando positivos para IAMCSST, e todos aqueles que são falsos negativos para IAMCSST, mas que apresentam achados clínicos, eletrocardiográficos e ecocardiográficos de OCA.
