# Supplementary material for: O Custo do Atraso: Disparidades Socioeconômicas e Falhas Diagnósticas no Tratamento de Infarto do Miocárdio com Oclusão
Source: Arq Bras Cardiol. 2024 Aug 2;121(7):e20240311. [Article in Portuguese] doi: 10.36660/abc.20240311 (PMC11341212; doi:10.36660/abc.20240311)
Supplement: Translation

# Translation: The Price of Delay: Socioeconomic Disparities and Diagnostic Oversights in Occlusion Myocardial Infarction Care

**DOI:** 10.36660/abc.20240311i

**Authors:** José Nunes de Alencar, Jesse T. T. McLaren

**Affiliations:** 1 Brazil Instituto Dante Pazzanese de Cardiologia, São Paulo, SP – Brazil; 2 Canada University Health Network - Emergency Department, Toronto, Ontario – Canada

**Keywords:** Myocardial Infarction, Acute Coronary Syndrome, Therapeutics

Acute myocardial infarction with acute coronary occlusion represents the most severe and urgent facet of acute coronary syndromes. Seminal studies have taught us that reestablishing coronary flow in patients with occluded arteries, whether through thrombolysis or primary angioplasty, can alter the natural history of the disease and significantly reduce mortality associated with this syndrome.^1,2^

The authors of “The Clinical and Economic Impact of Delayed Reperfusion Therapy: Real-World Evidence” provide compelling data from a wealthy metropolitan region in Brazil, concluding that each additional hour of delay in reperfusion therapy was associated with a 6.2% increase (95% confidence interval: 0.3% to 11.8%, p = 0.032) in the risk of in-hospital mortality.^3^ In addition, overall costs were 45% higher among individuals treated after 9 hours compared to those treated within the first 3 hours, which was mainly due to in-hospital costs (p = 0.005). Gioppatto et al.^3^ also note, from other studies, the financial impacts on patients and families due to delayed reperfusion, and they call for ST elevation myocardial infarction (STEMI) networks to address the uneven distribution of percutaneous coronary intervention (PCI)-capable centers.

However, we would like to add two issues to this equation and, in an exercise of imagination, extrapolate two groups of patients who would likely experience even worse outcomes than those described in this intriguing article.

Firstly, lower socioeconomic status, defined as low income and less than high school education, is a key determinant of inequalities in care, leading to poor health outcomes and reduced life expectancy. Studies conducted in Sweden, Finland, Canada, and the United States have shown that the prognosis of patients from lower socioeconomic status groups is worse following acute myocardial infarction as a result of inequalities in care.^4^ One study revealed that patients with myocardial infarction in the lowest median household income group in the United States were less likely to undergo coronary angiography and PCI.^5^ In other words, there is uneven access to care even within PCI centers, which exacerbates the uneven distribution of PCI-capable centers. As a result, socioeconomic gaps put patients at risk of delayed reperfusion, which creates further financial impacts on those with the least income. It is important to consider the fact that there are more severe economic disparities in Brazil than in the other countries mentioned in this paragraph.

There is a second group of patients who experience delayed reperfusion even within PCI-capable centers, with further delay based on geographic variation, namely, those who are false negatives within the current diagnostic paradigm of “myocardial infarction with and without ST-segment elevation (STEMI/NSTEMI).” In the current paradigm for diagnosing myocardial infarction, more than half of the patients with acute coronary occlusion (who therefore warrant immediate reperfusion of their occluded artery within door-to-balloon or door-to-needle times)^6^ do not exhibit ST-segment elevation and are diagnosed with NSTEMI.^7^ In other words, these patients have occlusion myocardial infarction (OMI), but are false negative STEMI, or STEMI(-)OMI. Under the current paradigm, these unfortunate patients experience delayed reperfusion at PCI-capable centers, often far beyond the 9 hours discussed in this article. Not only are these occlusions missed on arrival, but these false negatives are not recognized, even in hindsight, because their discharge diagnosis remains “NSTEMI.”^8^ As a result, these high-risk patients with STEMI(-)OMI are not included in STEMI databases and not considered a target for quality improvement.

Notably, Gioppatto et al.^3^ did not include these patients in their study, precisely because the authors chose to select only patients with positive test results (STEMI positive) rather than those with actual disease (acute coronary occlusion). Studies have shown that the outcomes of these patients continue to be worse than those who are fortunate enough to be true positive. In a 2018 meta-analysis of more than 60,000 patients with NSTEMI, 34% had an occluded culprit artery with lower ejection fraction, higher risk of cardiogenic shock, recurring myocardial infarction, and death.^9^ Herman et al.^10^ compared NSTEMI-OMI to STEMI-OMI and found a hazard ratio of 1.84 for 1-year mortality and 2.59 for 5-year mortality, with an absolute mortality difference of 15%. The mean time to intervention was 1.4 hours in the STEMI group and 16.3 hours in the NSTEMI-OMI group.^10^

Gioppatto et al.^3^ study marks a significant milestone in Brazilian cardiology by strongly highlighting the urgent need to reperfuse patients as soon as possible to avoid poor individual and collective outcomes. We add that the Brazilian cardiology community should also focus on two often overlooked groups with OMI who are denied timely and appropriate treatment: those with lower socioeconomic status who experience delayed reperfusion even when STEMI positive, and all those who are falsely STEMI negative but who have clinical, ECG, and echocardiographic findings of OMI.
